# EGCG Attenuates CA1 Neuronal Death by Regulating GPx1, NF-κB S536 Phosphorylation and Mitochondrial Dynamics in the Rat Hippocampus following Status Epilepticus

**DOI:** 10.3390/antiox12040966

**Published:** 2023-04-20

**Authors:** Ji-Eun Kim, Tae-Hyun Kim, Tae-Cheon Kang

**Affiliations:** Department of Anatomy and Neurobiology, Institute of Epilepsy Research, College of Medicine, Hallym University, Chuncheon 24252, Republic of Korea; jieunkim@hallym.ac.kr (J.-E.K.); hyun1028@hallym.ac.kr (T.-H.K.)

**Keywords:** epilepsy, c-Jun *N*-terminal kinase (JNK), mitochondrial dynamics, oxidative stress, seizure, U0126

## Abstract

Epigallocatechin-3-gallate (EGCG) is an antioxidant that directly scavenges reactive oxygen species (ROS) and inhibits pro-oxidant enzymes. Although EGCG protects hippocampal neurons from status epilepticus (SE, a prolonged seizure activity), the underlying mechanisms are not fully understood. As the preservation of mitochondrial dynamics is essential for cell viability, it is noteworthy to elucidate the effects of EGCG on impaired mitochondrial dynamics and the related signaling pathways in SE-induced CA1 neuronal degeneration, which are yet unclear. In the present study, we found that EGCG attenuated SE-induced CA1 neuronal death, accompanied by glutathione peroxidase-1 (GPx1) induction. EGCG also abrogated mitochondrial hyperfusion in these neurons by the preservation of extracellular signal-regulated kinase 1/2 (ERK1/2)–dynamin-related protein 1 (DRP1)-mediated mitochondrial fission, independent of c-Jun *N*-terminal kinase (JNK) activity. Furthermore, EGCG abolished SE-induced nuclear factor-κB (NF-κB) serine (S) 536 phosphorylation in CA1 neurons. ERK1/2 inhibition by U0126 diminished the effect of EGCG on neuroprotection and mitochondrial hyperfusion in response to SE without affecting GPx1 induction and NF-κB S536 phosphorylation, indicating that the restoration of ERK1/2–DRP1-mediated fission may be required for the neuroprotective effects of EGCG against SE. Therefore, our findings suggest that EGCG may protect CA1 neurons from SE insults through GPx1–ERK1/2–DRP1 and GPx1–NF-κB signaling pathways, respectively.

## 1. Introduction

Status epilepticus (SE, a prolonged seizure activity) is a life-threatening neurological emergency and carries a risk of major morbidity and mortality, which can have long-term consequences, including impaired cognition, neuronal death and aberrant neuronal networks [1,2]. Excitotoxicity induced by excessive Ca^2+^ influx through the *N*-methyl-D-aspartate receptor (NMDAR) is one of the pathological mechanisms for SE-induced neuronal death, which triggers programmed neuronal necrosis death [3,4].

**Figure 1:**
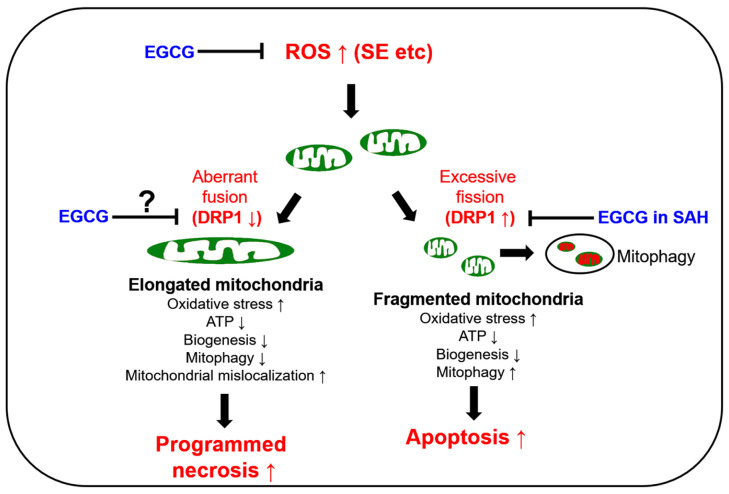
Scheme of the biochemical and molecular basis of mitochondrial functions in neuronal degeneration and EGCG-related changes in mitochondrial dynamics based on previous reports [12,13,14,15,16,17,18,19,20,23,24,25,26,27,28]. DRP1: dynamin-related protein 1; EGCG: epigallocatechin-3-gallate; ROS: reactive oxygen species; SAH: subarachnoid hemorrhage; SE: statue epilepticus; ↑: increase; ↓: decrease.

The preservation of mitochondrial mass, bioenergetic functions and reactive oxygen species (ROS) homoeostasis is required for neuronal survival because mitochondria produce most of the energy in the form of ATP to execute and maintain cell viability. Thus, mitochondrial dynamics such as fission (fragmentation) and fusion (elongation) are essential for the maintenance of the bioenergetics and Ca^2+^ buffering needs of neurons for neurotransmission. Mitochondrial fission plays an important role in mitochondrial motility, mitochondrial DNA inheritance, the regulation of mitochondrial size/shape, the distribution of mitochondria, mitochondria quality control and homeostasis and the removal of damaged mitochondria through mitophagy. However, excessive mitochondrial fission leads to neuronal death by impaired bioenergetics, ROS generation and the release of cytochrome *c* (a pro-apoptotic factor) from mitochondria [5,6,7,8]. Mitochondrial fusion is also critical to maintain the integrity of mitochondria. However, aberrant excessive mitochondrial fusion also evokes excessive ROS production by inhibiting mitochondrial respiration and induces ATP deficiency in peripheral sites by abolishing mitochondrial transports [3,9,10,11] (Figure 1).

GTPase dynamin-related protein 1 (DRP1) encoded on *DNM1L* is required for proper mitochondrial dynamics. DRP1 activity is oppositely regulated by two distinct serine (S) phosphorylation sites. DRP1 S616 phosphorylation facilitates mitochondrial fission, while S637 phosphorylation detaches DRP1 from mitochondria and subsequently inhibits fragmentation [12]. In humans, *DNM1L* mutations cause a mitochondrial fission deficiency (abnormal mitochondrial elongation) and then a sudden onset of refractory SE with very poor neurologic outcomes [13,14,15,16]. A mutation of the signal transducer and activator of transcription 2 (STAT2) also leads to intractable seizures, with DRP1 inactivation causing defective mitochondrial fission [17]. In experimental animal models, SE rapidly downregulated DRP1 expression, which evoked aberrant mitochondrial elongation leading to programmed necrotic neuronal death in CA1 pyramidal cells [3,18,19,20]. Although SE induces the apoptosis of parvalbumin (PV) interneurons, regulating the fast adaptation to repetitive spikes in the hilus of the dentate gyrus through DRP1-mediated excessive mitochondrial fragmentation [21], it is likely that at least in CA1 neurons, SE provokes programmed necrosis due to the hyperfusion of the mitochondrial network (aberrant mitochondrial elongation) by deregulating DRP1-mediated mitochondrial fission (Figure 1).

Epigallocatechin-3-gallate (EGCG) activates the non-integrin 67 kDa laminin receptor (67LR) [22,23]. EGCG also directly scavenges ROS, independent of 67LR [24,25], and acts as an antioxidant to inhibit pro-oxidant enzymes such as nicotinamide adenine dinucleotide phosphate (NADPH) oxidase and xanthine oxidase [26,27]. Indeed, EGCG showed a neuroprotective effect in an oxyhemoglobin (OxyHb)-induced rat subarachnoid hemorrhage (SAH) model by abrogating DRP1 upregulation and excessive mitochondrial fragmentation [28] (Figure 1). Unlike the SAH model, SE induced DRP1 downregulation and mitochondrial hyperfusion in the CA1 neurons of the dorsal hippocampus [3,18,19,20]. However, EGCG protected hippocampal pyramidal neurons from lithium chloride (LiCl)–pilocarpine-induced SE by inhibiting nuclear factor-κB (NF-κB) activity, although the effect of EGCG on mitochondrial dynamics has not been reported [29]. As oxidative stress is one of the common causes of neuronal damage induced by OxyHb-induced SAH [28], neuroinflammation [30,31] and SE [4,12] models, it is likely that the neuroprotective effects of EGCG may be relevant to oxidative stress-induced mitochondrial dynamics as well as NF-κB inhibition. Indeed, EGCG increases glutathione peroxidase-1 (GPx1), which plays an important role in the reduction of the H_2_O_2_ level as a cofactor of glutathione (GSH), and GPx1 downregulation is involved in SE-induced CA1 neuronal death [32,33,34,35,36]. Furthermore, EGCG attenuates the increment of NADPH oxidase and superoxide dismutase (SOD) activities, but not H_2_O_2_ production [37]. Considering that oxidative stress induces neuronal death caused by impaired mitochondrial dynamics [38,39,40] and EGCG improves ROS-induced mitochondrial dysfunctions [28,41,42], it is likely, therefore, that EGCG-mediated GPx1 upregulation may attenuate SE-induced CA1 neuronal death by affecting impaired mitochondrial dynamics as well as NF-κB activation, which is still elusive. Thus, the present study was conducted to investigate the effects of EGCG on CA1 neuronal degeneration, aberrant mitochondrial hyperfusion, NF-κB phosphorylation and GPx1 downregulation induced by SE.

Here, we demonstrate that EGCG attenuated SE-induced CA1 neuronal death and aberrant mitochondrial elongation in CA1 neurons. These neuroprotective effects of EGCG were relevant to GPx1 upregulation in CA1 neurons, which prevented mitochondrial hyperfusion by the preservation of extracellular signal-regulated kinase 1/2 (ERK1/2)-mediated DRP1 S616 phosphorylation, independent of the c-Jun *N*-terminal kinase (JNK) signaling pathway. EGCG-induced GPx1 upregulation also inhibited NF-κB S536 phosphorylation in CA1 neurons following SE. ERK1/2 inhibition by U0126 diminished the effect of EGCG on neuroprotection and mitochondrial hyperfusion without affecting GPx1 induction and NF-κB S536 phosphorylation. Therefore, our findings suggest that EGCG-induced GPx1 upregulation may protect CA1 neurons from SE insults through the ERK1/2–DRP1-mediated restoration of impaired mitochondrial elongation as well as the inhibition of NF-κB S536 phosphorylation.

## 2. Materials and Methods

### 2.1. Experimental Animals and Chemicals

Seventy male Sprague-Dawley rats (200–220 g; Daehan biolink, Umseong, Chungcheongbuk-do, Republic of Korea) were used in this experiment. Twenty-eight rats were used for the control group and forty-two rats were used for the experimental groups, respectively. The rats were housed under a 12 h dark/light cycle with a standard rodent diet and water. All experimental procedures were formally approved by the Institutional Animal Care and Use Committee of Hallym University (Hallym 2021-3; approval date, 17 May 2021). All reagents were obtained from Sigma-Aldrich (St. Louis, MO, USA), except as noted.

### 2.2. Surgical Procedures and SE Induction

Rats were implanted with an infusion needle (brain infusion kit 1; Alzet, Cupertino, CA, USA) into the right lateral ventricle (coordinates: 1 mm posterior; 1.5 mm lateral; 3.5 mm depth) under isoflurane anesthesia (3% induction, 1.5–2% for surgery and 1.5% maintenance in a 65:35 mixture of N_2_O:O_2_), followed by connecting an osmotic pump (1007D; Alzet, Cupertino, CA, USA) containing (1) a vehicle (*n* = 28), (2) EGCG (50 μM) (*n* = 28) or (3) EGCG (50 μM) + U0126 (25 μM) (*n* = 14). In pilot and previous studies [23], each treatment did not evoke neurological adverse effects or alter the seizure susceptibility and its severity in response to pilocarpine. Two days after surgery, the rats were injected with LiCl (127 mg/kg, i.p.). The next day, 20 min before pilocarpine administration, the animals received atropine methylbromide (5 mg/kg i.p.). Thereafter, the animals were given pilocarpine (30 mg/kg, i.p.; *n* = 42). Two hours after SE onset, diazepam (Valium; Hoffmann-la Roche, Neuilly-sur-Seine, France; 10 mg/kg, i.p.) was injected to cease seizure activity, and repeated as needed. Control animals (*n* = 28) were injected with saline in place of pilocarpine.

### 2.3. Western Blot

Three days after SE, the animals were decapitated under urethane anesthesia (1.5 g/kg, i.p.). The hippocampus of each animal (*n* = 7 rats in each group) was rapidly collected. After homogenization with a lysis buffer containing a protease inhibitor cocktail (Roche Applied Sciences, Branford, CT, USA) and a phosphatase inhibitor cocktail (PhosSTOP^®^; Roche Applied Science, Branford, CT, USA), protein concentrations were determined using a Micro BCA Protein Assay Kit (Pierce Chemical, Rockford, IL, USA). An equal amount (10 μg) of each sample was loaded onto Bis-Tris sodium dodecyl sulfate-poly-acrylamide electrophoresis gel (SDS-PAGE). The proteins were separated by electrophoresis and transferred to polyvinylidene fluoride membranes. The membranes were then immunoblotted using a primary antibody (Table 1). Rabbit anti-β-actin was used as an internal reference for data normalization. An ECL Kit (GE Healthcare Korea, Seoul, South Korea) was used to visualize the signals, and the proteins were analyzed using an ImageQuant LAS4000 system (GE Healthcare Korea, Seoul, South Korea). The phosphoprotein:total protein ratio was described as the phosphorylation ratio.

### 2.4. Immunohistochemistry and Mitochondrial Morphometry

Three days after SE, animals were anesthetized with urethane anesthesia (1.5 g/kg, i.p.) and perfused through the left ventricle with normal saline followed by 4% paraformaldehyde in a 0.1 M phosphate buffer (PB; pH 7.4). The brains were post-fixed in the same fixative, cryoprotected with 30% sucrose overnight and cut at a thickness of 30 μm using a cryostat. Hippocampal tissues approximately −3.0~−3.6 mm from the bregma were selected based on the rat brain from stereotaxic coordinates [43]. After blocking with 3% bovine serum albumin in PBS for 30 min at room temperature, the sections were reacted with a cocktail solution containing primary antibodies (Table 1) overnight at room temperature, followed by incubation with Cy2- or Cy3-conjugated secondary antibodies. A negative control test was performed with pre-immune serum substituted for the primary antibody.

Pilocarpine-induced SE results in the most pronounced CA1 neuronal loss of the dorsal hippocampus of adult rats [44], while it further degenerates neurons in the ventral hippocampus of immature and young rats [45,46]. Furthermore, aberrant mitochondrial elongation was predominantly detected in the SE-vulnerable CA1 neurons of the dorsal hippocampus in our previous studies [3,19,20,21]. Therefore, the fluorescent intensities and mitochondrial morphometry were analyzed from five CA1 neurons randomly selected in the pyramidal cell layer of the dorsal hippocampus (five sections from each animal; *n* = 7 rats in each group) using AxioVision Rel. 4.8 software (Carl Zeiss Korea, Seoul, Republic of Korea) and ImageJ software. To conduct mitochondrial morphometry, the mitochondrial elongation index (area-weighted form factor = perimeter^2^/4π) and mitochondrial network aggregation (cumulative area:perimeter ratio = Σarea/Σperimeter) in these neurons were calculated using ImageJ software. The mitochondrial elongation index indicates the increased mitochondrial length (mitochondrial elongation) by the transition from a punctiform to an elongated shape. In addition, mitochondrial network aggregation (cumulative area:perimeter ratio) indicates the transition from elongated, isolated mitochondria to a reticular network or the aggregation of interconnected mitochondria [47,48].

### 2.5. Fluoro-Jade B (FJB) Staining

For the quantification of neuronal degeneration, some tissue sections (*n* = 4–5 sections in each animal) were mounted on a glass slide and reacted with 0.06% KMnO_4_, followed by 0.001% FJB solution (Histo-Chem Inc., Jefferson, AR, USA). The number of FJB-positive neurons was measured using AxioVision Rel. 4.8.

### 2.6. Data Analysis

Data were analyzed using the Mann–Whitney test or Kruskal–Wallis test followed by a Dunn–Bonferroni post hoc comparison. A *p*-value less than 0.05 was considered to be significant.

## 3. Results

### 3.1. EGCG Attenuates SE-Induced CA1 Neuronal Death

**Figure 2:**
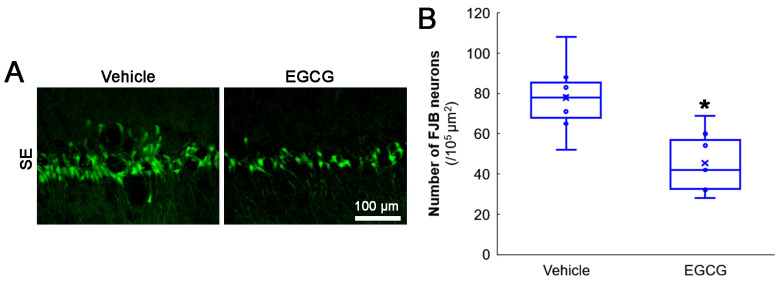
Effects of EGCG on SE-induced CA1 neuronal damage. EGCG diminished the number of FJB-positive-degenerating CA1 neurons in SE group. (**A**) Representative FJB-positive-degenerating CA1 neurons following SE. (**B**) Quantification of the number of FJB-positive neurons (* *p* < 0.05 vs. vehicle-treated animals; *n* = 7 rats, respectively).

First, we evaluated the effects of EGCG on SE-induced CA1 neuronal death. Compared with the vehicle, EGCG effectively attenuated CA1 neuronal death induced by SE (*z* = 2.619 and *p* = 0.009, *n* = 7 rats, respectively; Mann–Whitney test; Figure 2A,B).

### 3.2. EGCG Diminishes SE-Induced GPx1 Downregulation in CA1 Neurons

SE leads to GPx1 downregulation in CA1 neurons [3,19]. As EGCG protects against oxidative stress by acting as a natural free radical scavenger and enhances GPx1 expression [35,49], we investigated whether EGCG affected GPx1 expression in CA1 neurons following SE.

**Figure 3:**
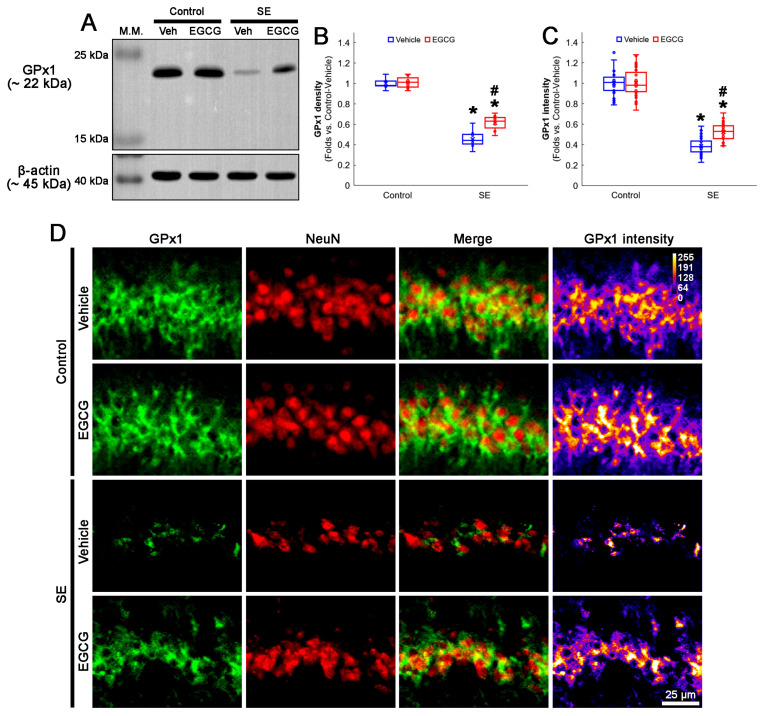
Effects of EGCG on GPx1 expression following SE. Western blot data showed that GPx1 expression decreased in SE group, compared with control animals. In control animals, EGCG did not affect GPx1 expression. EGCG attenuated the reduced GPx1 expression in SE group (**A**–**C**). Immunofluorescent study also revealed that GPx1 expression was reduced in CA1 neurons of SE group, compared with control animals. In control animals, EGCG did not affect GPx1 expression in CA1 neurons. EGCG attenuated the decreased GPx1 expression in the CA1 neurons of SE group (**D**). (**A**) Representative Western blot of GPx1 in the whole hippocampus. (**B**) Quantification of GPx1 protein level based on Western blot data (* and # *p* < 0.05 vs. control and vehicle-treated rats; *n* = 7 rats, respectively). (**C**) Quantification of GPx1 intensity in CA1 neurons (* and # *p* < 0.05 vs. control and vehicle-treated animals; *n* = 35 cells in 7 rats, respectively). (**D**) Representative photos of GPx1 expression and its intensity.

In the control animals, EGCG did not affect the GPx1 protein level in the hippocampus (Figure 3A,B and Figure S1). SE reduced the GPx1 protein level to 0.46-fold of the control vehicle-treated animal level in the hippocampus (*p* < 0.001; Dunn–Bonferroni post hoc test), which was enhanced to 0.61-fold of the control vehicle-treated animal level by EGCG (*p* = 0.041; Dunn–Bonferroni post hoc test; *χ^2^*_(3)_ = 22.03; *p* < 0.001; *n* = 7 rats, respectively; Kruskal–Wallis test; Figure 3A,B and Figure S1). Compatible with the Western blot data, SE decreased the GPx1 fluorescent intensity to 0.39-fold of the control vehicle-treated animal level in the CA1 neurons (*p* < 0.001; Dunn–Bonferroni post hoc test), which was mitigated by EGCG (*p* < 0.001; Dunn–Bonferroni post hoc test; *χ^2^*_(3)_ = 111.685; *p* < 0.001; *n* = 35 cells in 7 rats, respectively; Kruskal–Wallis test; Figure 3C,D). These findings indicated that EGCG may prevent GPx1 downregulation from SE, which would ameliorate CA1 neuronal death.

### 3.3. EGCG Ameliorates SE-Induced Aberrant Mitochondrial Elongation in CA1 Neurons

GPx1 protects neurons from oxidative stress, which evokes neuronal degeneration mediated by impaired mitochondrial dynamics [32,33,38,39,40]. Thus, we investigated whether EGCG influenced the mitochondrial dynamics in CA1 neurons following SE.

**Figure 4:**
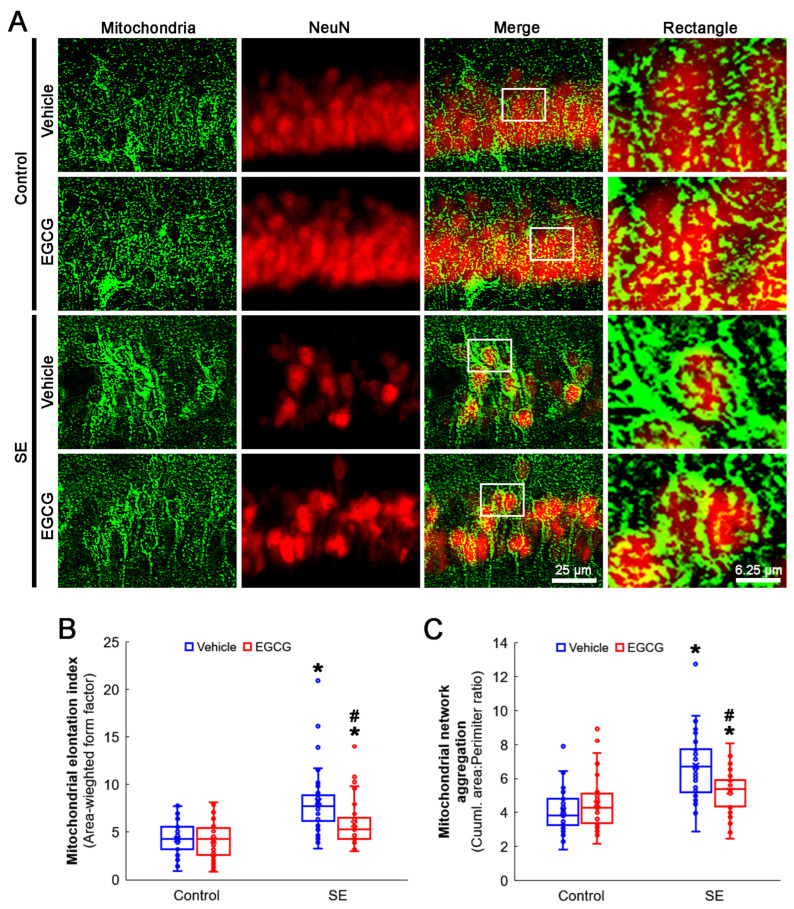
Effects of EGCG on mitochondrial dynamics in CA1 neurons following SE. Compared with control animals, mitochondria showed swollen, elongated and complex shapes in SE group. Mitochondria were also transformed into a reticular network or an aggregation of interconnected mitochondria in the CA1 neurons in SE group. In control animals, EGCG did not affect mitochondrial shapes in CA1 neurons, compared with vehicle-treated animals. However, EGCG attenuated SE-induced morphological changes in mitochondria. (**A**) Representative photos of mitochondria and CA1 neurons (NeuN, a neuronal marker). Rectangle indicates the high-magnification photos in rectangles on merge panel. (**B**) Quantification of mitochondrial elongation index in CA1 neurons (* and # *p* < 0.05 vs. control and vehicle-treated animals; *n* = 35 cells in 7 rats, respectively). (**C**) Quantification of mitochondrial network aggregation in CA1 neurons (* and # *p* < 0.05 vs. control and vehicle-treated animals; *n* = 35 cells in 7 rats, respectively).

SE elongated the mitochondrial length in CA1 neurons (*p* < 0.001; Dunn–Bonferroni post hoc test). Although EGCG did not alter the mitochondrial length in the control rats, it effectively diminished SE-induced elongation (*p* = 0.001; Dunn–Bonferroni post hoc test; *χ^2^*_(3)_ = 42.741; *p* = 0.001; *n* = 35 cells in 7 rats, respectively; Kruskal–Wallis test; Figure 4A,B). SE also led to mitochondrial aggregation in CA1 neurons (*p* < 0.001; Dunn–Bonferroni post hoc test). EGCG ameliorated aberrant mitochondrial aggregation in CA1 neurons (*p* = 0.022; Dunn–Bonferroni post hoc test; *χ^2^*_(3)_ = 41.646; *p* < 0.001; *n* = 35 cells in 7 rats, respectively; Kruskal–Wallis test), while it could not affect the mitochondrial reticular networks in the control animals (Figure 4A,C). These findings indicated that SE may lead to mitochondrial hyperfusion in CA1 neurons, which would be inhibited by EGCG.

### 3.4. EGCG Attenuates a Decrease in DRP1 Expression and Its S616 Phosphorylation in CA1 Neurons

**Figure 5:**
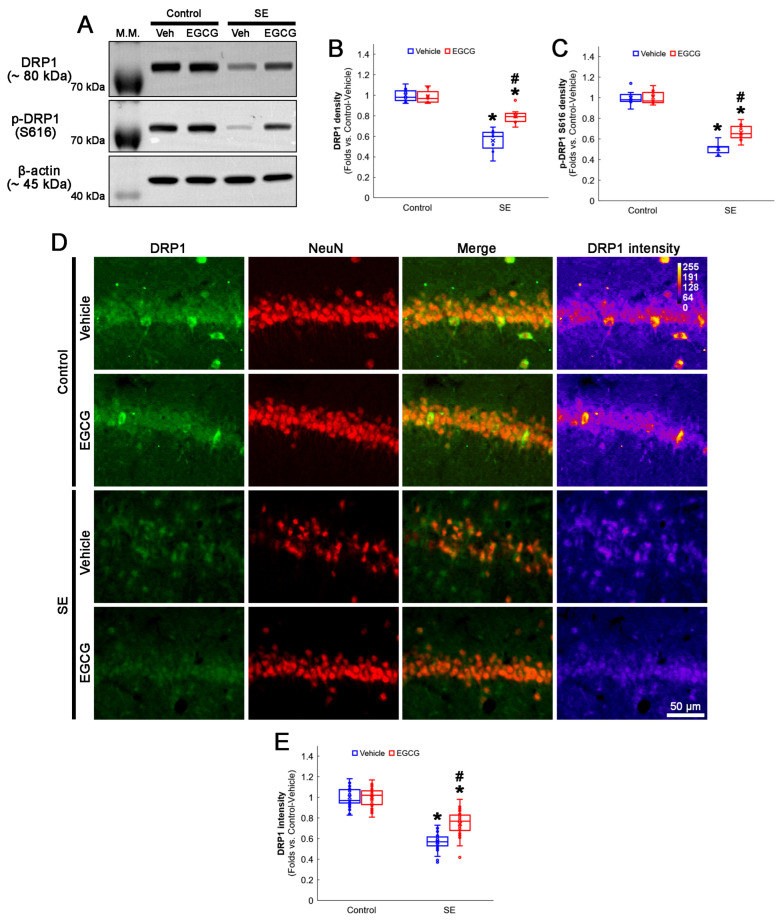
Effects of EGCG on DRP1 and its S616 phosphorylation following SE. Western blot data showed that DRP1 expression and its S616 phosphorylation decreased in SE group, compared with control animals. In control animals, EGCG did not affect DRP1 expression and its S616 phosphorylation level. EGCG attenuated a decrease in DRP1 expression and its S616 phosphorylation in SE group (**A**–**C**). Immunofluorescent study also revealed that DRP1 expression was reduced in CA1 neurons of SE group, compared with control animals. In control animals, EGCG did not affect DRP1 expression in CA1 neurons. EGCG attenuated a decrease in DRP1 expression in CA1 neurons of SE group (**D**,**E**). (**A**) Representative Western blot of DRP1 and its S616 phosphorylation in the whole hippocampus. (**B**) Quantification of DRP1 protein level based on Western blot data (* and # *p* < 0.05 vs. control and vehicle-treated rats; *n* = 7 rats, respectively). (**C**) Quantification of DRP1 S616 level based on Western blot data (* and # *p* < 0.05 vs. control and vehicle-treated rats; *n* = 7 rats, respectively). (**D**) Representative photos of DRP1 expression and its intensity. (**E**) Quantification of DRP1 intensity in CA1 neurons (* and # *p* < 0.05 vs. control and vehicle-treated animals; *n* = 35 cells in 7 rats, respectively).

As DRP1 expression and its S616 phosphorylation are significantly decreased in CA1 neurons 3 days after SE [3,19], we validated the effect of EGCG on DRP1 expression and its S616 phosphorylation following SE. The Western blot data showed that EGCG did not affect DRP1 expression and its S616 phosphorylation in the hippocampus of the control animals (Figure 5A–C). Following SE, the total DRP1 protein level was reduced to 0.56-fold of the control vehicle-treated animal level in the hippocampus (*p* < 0.001; Dunn–Bonferroni post hoc test), which increased to 0.8-fold of the control vehicle-treated animal level by EGCG (*p* = 0.01; Dunn–Bonferroni post hoc test; *χ^2^*_(3)_ = 21.275; *p* < 0.001; *n* = 7 rats, respectively; Kruskal–Wallis test; Figure 5A,B and Figure S2). Furthermore, the DRP1 S616 phosphorylation level was 0.51-fold of the control vehicle-treated animal level in the hippocampus (*p* < 0.001; Dunn–Bonferroni post hoc test). EGCG enhanced it to 0.66-fold of the control vehicle-treated animal level (*p* = 0.016; Dunn–Bonferroni post hoc test; *χ^2^*_(3)_ = 21.275; *p* < 0.001; *n* = 7 rats, respectively; Kruskal–Wallis test; Figure 5A,C and Figure S2). A double immunofluorescent study also revealed that SE decreased the DRP1 expression in CA1 neurons (*p* < 0.001; Dunn–Bonferroni post hoc test), which was attenuated by EGCG (*p* < 0.001; Dunn–Bonferroni post hoc test; *χ^2^*_(3)_ = 105.805; *p* < 0.001; *n* = 35 cells in 7 rats, respectively; Kruskal–Wallis test; Figure 5D,E). These findings indicated that EGCG may ameliorate SE-induced CA1 neuronal death by facilitating DRP1-mediated mitochondrial fission.

### 3.5. EGCG Enhances ERK1/2 but Not JNK Phosphorylation in CA1 Neurons following SE

The ERK1/2 and JNK signaling pathways regulate DRP1 S616 phosphorylation [50,51]. Furthermore, SE diminishes the ERK1/2 and JNK phosphorylation level (activity), while it does not affect their total protein levels in the rat hippocampus [20]. Therefore, it is likely that EGCG may ameliorate SE-induced CA1 neuronal death by facilitating DRP1-mediated mitochondrial fissions through the ERK and/or JNK pathways. To confirm this, we explored the effects of EGCG on ERK1/2 and JNK activities (phosphorylation) following SE.

**Figure 6:**
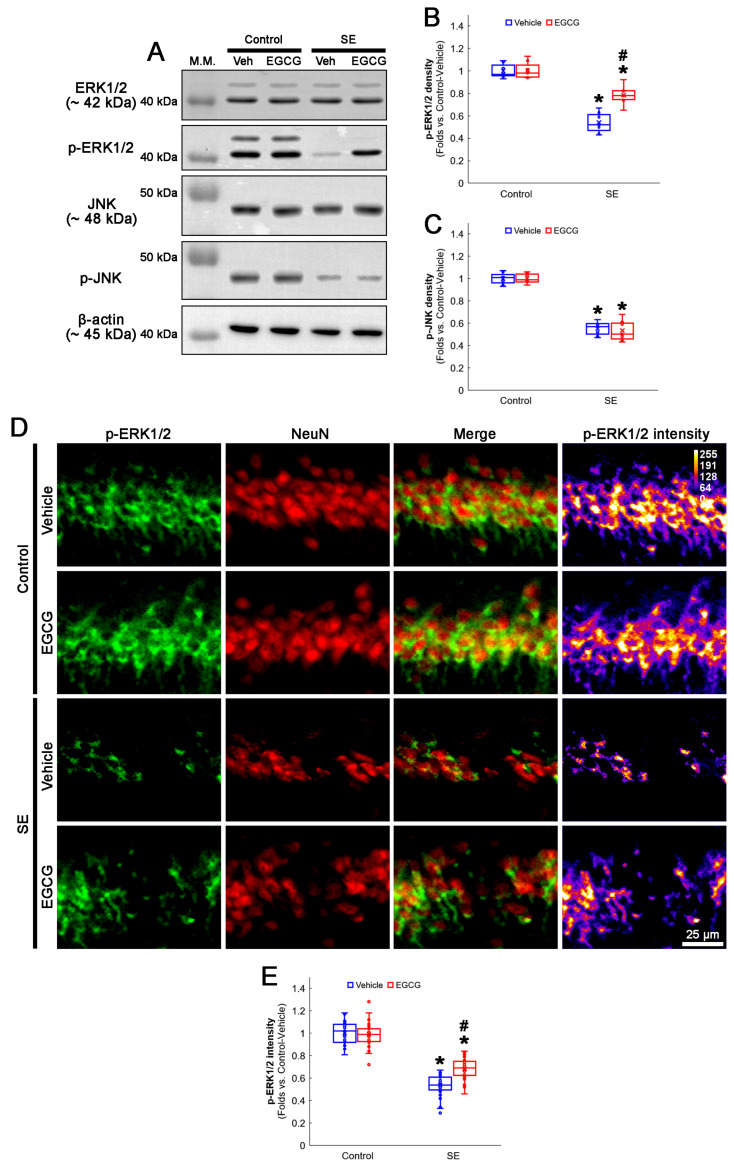
Effects of EGCG on ERK1/2 and JNK activities following SE. Western blot data showed that ERK1/2 and JNK phosphorylation decreased in SE group without affecting their total protein levels. In control animals, EGCG did not affect ERK1/2 and JNK phosphorylation. EGCG attenuated decreased ERK1/2 but not JNK phosphorylation in SE group (**A**–**C**). Immunofluorescent study revealed that ERK1/2 phosphorylation was reduced in CA1 neurons of SE group, compared with control animals. In control animals, EGCG did not affect ERK1/2 phosphorylation in CA1 neurons. EGCG attenuated a decrease in ERK1/2 phosphorylation in the CA1 neurons (**D**,**E**) of SE group. (**A**) Representative Western blot of ERK1/2, JNK and their phosphorylation in the whole hippocampus. (**B**) Quantification of ERK phosphorylation level based on Western blot data (* and # *p* < 0.05 vs. control and vehicle-treated rats; *n* = 7 rats, respectively). (**C**) Quantification of JNK phosphorylation level based on Western blot data (* *p* < 0.05 vs. control rats; *n* = 7 rats, respectively). (**D**) Representative photos of phospho (p)-ERK1/2 level and its intensity. (**E**) Quantification of p-ERK1/2 intensity in CA1 neurons (* and # *p* < 0.05 vs. control and vehicle-treated animals; *n* = 35 cells in 7 rats, respectively).

In the control animals, EGCG did not change the ERK1/2 and JNK total protein and phosphorylation levels in the hippocampus (Figure 6A–C and Figure S2). SE diminished the phospho (p)-ERK1/2 level to 0.54-fold of the control vehicle-treated animal level in the hippocampus (*p* < 0.001; Dunn–Bonferroni post hoc test), which was enhanced to 0.78-fold of the control vehicle-treated animal level by EGCG (*p* = 0.009; Dunn–Bonferroni post hoc test; *χ^2^*_(3)_ = 22.627; *p* < 0.001; *n* = 7 rats, respectively; Kruskal–Wallis test; Figure 6A,B and Figure S2). The p-JNK level was also decreased to 0.55-fold of the control vehicle-treated animal level in the hippocampus (*p* < 0.001; Dunn–Bonferroni post hoc test; *χ^2^*_(3)_ = 20.362; *p* < 0.001; *n* = 7 rats, respectively; Kruskal–Wallis test; Figure 6A,C and Figure S2). However, EGCG did not affect the decreased p-JNK level following SE (Figure 6A,C and Figure S2). A double immunofluorescent study also revealed that SE downregulated the p-ERK1/2 level in CA1 neurons (*p* < 0.001; Dunn–Bonferroni post hoc test), which was attenuated by EGCG (*p* < 0.001; Dunn–Bonferroni post hoc test; *χ^2^*_(3)_ = 109.938; *p* < 0.001; *n* = 35 cells in 7 rats, respectively; Kruskal–Wallis test; Figure 6D,E). These findings indicated that EGCG may facilitate mitochondrial fission by enhancing ERK1/2 but not JNK-mediated DRP1 S616 phosphorylation.

### 3.6. EGCG Ameliorates NF-κB S536 Phosphorylation in CA1 Neurons following SE

**Figure 7:**
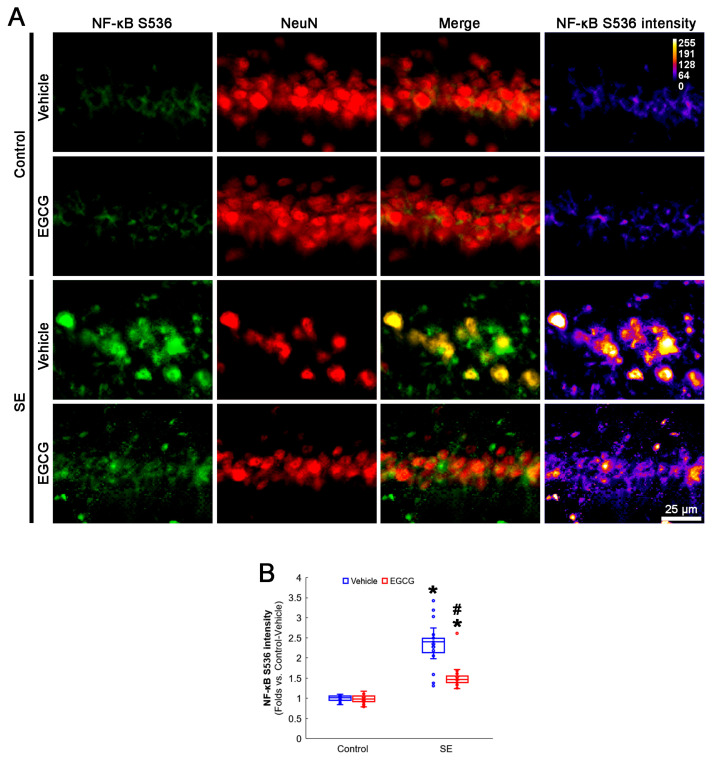
Effects of EGCG on NF-κB S536 phosphorylation in CA1 neurons following SE. Compared with control animals, NF-κB S536 phosphorylation was augmented in CA1 neurons of SE group. In control animals, EGCG did not affect NF-κB S536 phosphorylation level. EGCG attenuated NF-κB S536 phosphorylation in CA1 neurons of SE group. (**A**) Representative photos of NF-κB S536 phosphorylation level and its intensity. (**B**) Quantification of NF-κB S536 intensity in CA1 neurons (* and # *p* < 0.05 vs. control and vehicle-treated animals; *n* = 35 cells in 7 rats, respectively).

NF-κB S536 phosphorylation is relevant to the vulnerability of CA1 neurons in response to SE [52,53]. EGCG mitigates SE-induced neuronal damage by inhibiting the NF-κB signaling pathway [29]. Interestingly, mitochondrial fission and the NF-κB signaling pathway reciprocally regulate each other [54,55]. As GPx1 inhibits NF-κB S536 phosphorylation [56,57], it is likely that EGCG protects CA1 neurons from SE by affecting NF-κB S536 phosphorylation. Consistent with previous studies [52,53], the present study revealed that NF-κB S536 phosphorylation was very weakly observed in CA1 neurons. Following SE, the NF-κB S536 phosphorylation level was significantly enhanced in these neurons (*p* < 0.001; Dunn–Bonferroni post hoc test). In addition, EGCG effectively ameliorated the SE-induced enhancement of NF-κB S536 phosphorylation in CA1 neurons (*p* < 0.001; Dunn–Bonferroni post hoc test; *χ^2^*_(3)_ = 111.461; *p* < 0.001; *n* = 35 cells in 7 rats, respectively; Kruskal–Wallis test; Figure 7A,B). Considering the inhibitory effect of EGCG on SE-induced mitochondrial hyperfusion in the present study (Figure 4), these data indicated that enhanced NF-κB S536 phosphorylation may not be involved in aberrant mitochondrial elongation in CA1 neurons. Therefore, our findings suggested that EGCG may ameliorate SE-induced CA1 neuronal degeneration by abrogating NF-κB S536 phosphorylation, independent of mitochondrial dynamics.

### 3.7. ERK1/2 Inhibition Abrogates the Effect of EGCG on SE-Induced CA1 Neuronal Degeneration

**Figure 8:**
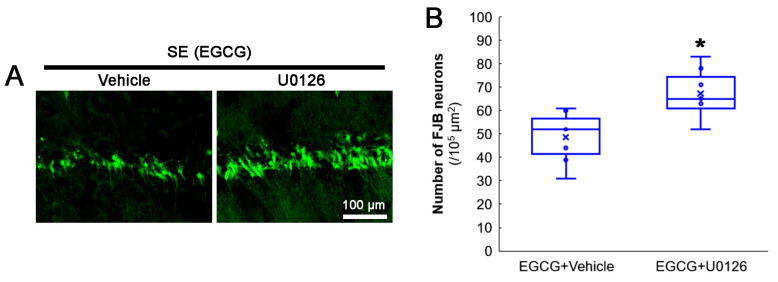
Effects of ERK1/2 inhibition by U0126 co-treatment on SE-induced neuronal damage in CA1 neurons of EGCG-treated rats. Compared with the vehicle, U0126 co-treatment abolished the protective effect of EGCG on SE-induced neuronal degeneration. (**A**) Representative FJB-positive-degenerating CA1 neurons following SE. (**B**) Quantification of the number of FJB-positive neurons (* *p* < 0.05 vs. vehicle co-treatment; *n* = 7 rats, respectively).

The protective mechanism of EGCG against the oxidative burden includes ERK1/2 activation [58,59,60,61]. Indeed, the present study demonstrated that EGCG increased ERK1/2 phosphorylation and inhibited aberrant mitochondrial elongation in CA1 neurons. Therefore, we applied a U0126 (an ERK1/2 inhibitor) co-treatment with EGCG prior to the SE induction to confirm the role of ERK1/2 in the neuroprotective effect of EGCG on SE-induced neuronal death. Compared with the vehicle, the U0126 co-treatment diminished the neuroprotective effect of EGCG on SE-induced CA1 neurons (*z* = 2.43; *p* = 0.015; *n* = 7 rats, respectively; Mann–Whitney test; Figure 8A,B). These findings indicated that ERK1/2 activation may be required for the neuroprotective effect of EGCG against SE.

### 3.8. ERK1/2 Inhibition Abrogates the Effect of EGCG on Mitochondrial Hyperfusion

**Figure 9:**
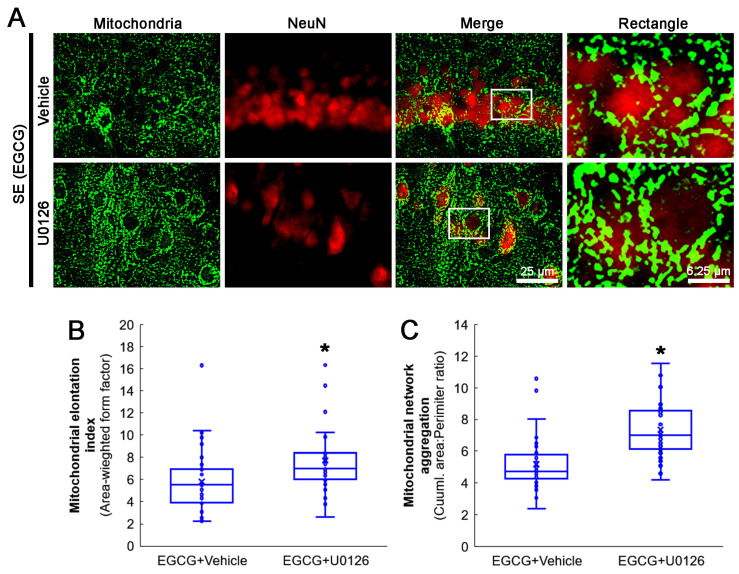
Effects of ERK1/2 inhibition by U0126 co-treatment on mitochondrial dynamics in CA1 neurons of EGCG-treated rats following SE. Compared with the vehicle, U0126 co-treatment increased the mitochondrial length and the formation of mitochondrial network aggregation in CA1 neurons. (**A**) Representative photos of mitochondria and CA1 neurons (NeuN, a neuronal marker). Rectangle indicates the high-magnification photos in rectangles on merge panel. (**B**) Quantification of mitochondrial elongation index in CA1 neurons (* *p* < 0.05 vs. vehicle co-treatment; *n* = 35 cells in 7 rats, respectively). (**C**) Quantification of mitochondrial network aggregation in CA1 neurons (* *p* < 0.05 vs. vehicle co-treatment; *n* = 35 cells in 7 rats, respectively).

Next, we investigated whether the U0126 co-treatment influenced the effect of EGCG on aberrant mitochondrial elongation in CA1 neurons following SE. Compared with the vehicle, the U0126 co-treatment increased the mitochondrial elongation index (*z* = 3.172; *p* = 0.002; *n* = 35 cells in 7 rats, respectively; Mann–Whitney test) and mitochondrial aggregation (*z* = 4.939; *p* < 0.001; *n* = 35 cells in 7 rats, respectively; Mann–Whitney test; Figure 9A–C) in the CA1 neurons of EGCG-treated rats following SE. These findings indicated that EGCG–ERK1/2 activation may restore the SE-induced impairment of mitochondrial fission in CA1 neurons.

### 3.9. U0126 Co-Treatment Inhibits EGCG-Induced DRP1 S616 Phosphorylation without Affecting GPx1 Induction and NF-κB S536 Phosphorylation following SE

**Figure 10:**
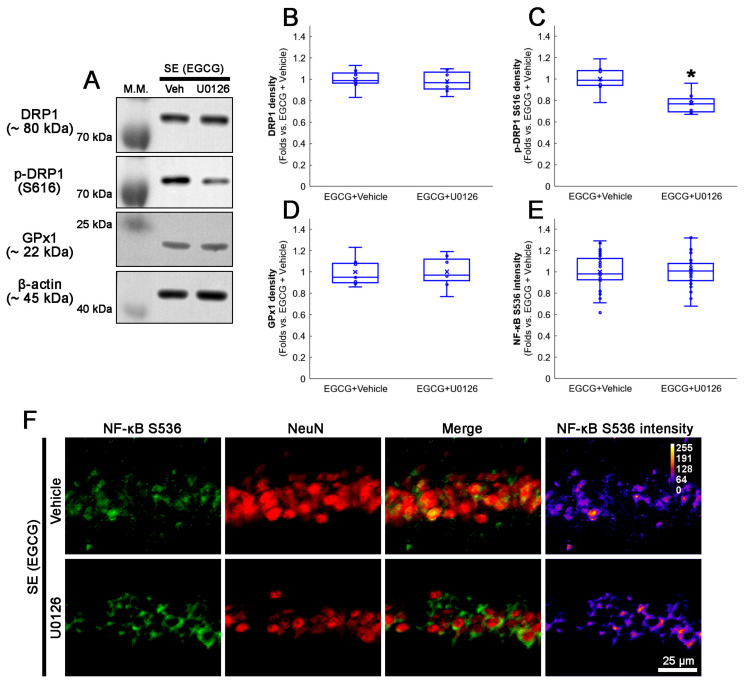
Effects of U0126 co-treatment on DRP1 S616, GPx1 and NF-κB S536 levels in EGCG-treated rats following SE. Western blot data showed that U0126 co-treatment diminished DRP1 S616 phosphorylation without affecting GPx1 expression and NF-κB S536 phosphorylation level in EGCG-treated rats following SE (**A**–**E**). Immunofluorescent study revealed that U0126 co-treatment did not affect NF-κB S536 phosphorylation level in EGCG-treated rats following SE, compared with vehicle. (**A**) Representative Western blot of DRP1, DRP1 S616 and GPx1 in the whole hippocampus. (**B**–**D**) Quantification of DRP1, DRP1 S616 and GPx1 levels based on Western blot data (* *p* < 0.05 vs. vehicle co-treatment; *n* = 7 rats, respectively). (**E**) Quantification of NF-κB S536 intensity in CA1 neurons (* *p* < 0.05 vs. control and vehicle-treated animals; *n* = 35 cells in 7 rats, respectively). (**F**) Representative photos of NF-κB S536 phosphorylation level and its intensity.

We also confirmed whether a U0126 co-treatment affected the activation of the ERK1/2–DRP1 signaling pathway induced by EGCG. As GPx1 activates ERK1/2 phosphorylation [62,63,64], the effect of a U0126 co-treatment on EGCG-induced GPx1 upregulation was investigated. Compared with the vehicle, the U0126 co-treatment did not affect the total DRP1 protein level (Figure 10A–C and Figure S4). However, EGCG decreased DRP1 S616 phosphorylation in the hippocampus (*z* = 2.492; *p* = 0.013; *n* = 7 rats, respectively; Mann–Whitney test; Figure 10A–C and Figure S4). The U0126 co-treatment did not influence the GPx1 expression in the hippocampus of EGCG-treated rats following SE (Figure 10A,D and Figure S4). An immunofluorescent study also revealed that the U0126 co-treatment did not affect NF-κB S536 phosphorylation in the CA1 neurons of EGCG-treated rats following SE (Figure 10E,F). Thus, our findings indicated that EGCG-mediated ERK1/2 activation may facilitate DRP1-mediated mitochondrial dynamics without affecting GPx1 induction as well as NF-κB S536 phosphorylation.

## 4. Discussion

Oxidative stress and mitochondrial dysfunction are critical events contributing to the etiology of neurological disorders. Indeed, a ROS-induced imbalance of the mitochondrial dynamic network is involved in neurodegeneration in Parkinson’s disease, Huntington’s disease, Alzheimer’s disease and epilepsy [12]. Therefore, the restoration of the antioxidant defense system and mitochondrial functions/dynamics could be of importance to counteract such pathological conditions. Considering the antioxidative properties of EGCG to protect neurons against various neurological diseases [24,25,26,27,28,29], it is likely that EGCG may inhibit oxidative stress, which results in mitochondrial dysfunctions, recovering impaired mitochondrial dynamics that generate further ROS [12]. However, the underlying mechanisms of EGCG remain poorly understood.

EGCG attenuates mitochondrial swelling, mitochondrial crystal dissolution and electron density induced by oxidative stress [65], and restores the mitochondrial membrane potential, mitochondrial function and ATP production in neurons and astrocytes following various insults [66,67,68]. Indeed, EGCG accumulates in the mitochondria and acts locally as a free radical scavenger [49]. Furthermore, EGCG protects neurons from oxidative stress through ERK1/2 activation [58,59,60,61]. In the present study, we found that EGCG ameliorated SE-induced CA1 neuronal degeneration by restoring ERK1/2–DRP1-mediated mitochondrial fission. The present data also demonstrated that EGCG ameliorated SE-induced GPx1 downregulation in CA1 neurons. GPx1 removes H_2_O_2_ by acting as a cofactor of GSH [32,33], and EGCG increases the GSH level that upregulates GPx1 expression in CA1 neurons [34,36]. Furthermore, GPx1 activates ERK1/2 phosphorylation [62,63,64]. Therefore, our findings indicated that the antioxidative activity of EGCG may protect CA1 neurons from impaired mitochondrial elongation induced by ROS.

The maintenance of mitochondrial fission mediated by DRP1 S616 phosphorylation is essential for the viability of CA1 neurons following SE [3,4,18,19,20]. DRP1 S616 phosphorylation is regulated by ERK1/2 and JNK [50,51]. SE-induced CA1 neuronal death is relevant to decreases in ERK1/2 and JNK phosphorylation [20]. Furthermore, EGCG relieves stress-induced ERK1/2 inactivation [59,60]. Consistent with these previous studies, the present data demonstrated that EGCG augmented GPx1–ERK1/2-mediated DRP1 S616 phosphorylation, accompanied by an inhibition of mitochondrial hyperfusion/aggregation. On the other hand, 2-cyano-3,12-dioxo-oleana-1,9(11)-dien-28-oic acid methyl ester (CDDO-Me, a triterpenoid antioxidant) attenuated SE-induced CA1 neuronal death by enhancing ERK1/2 and JNK phosphorylation [20]. In the present study, however, EGCG did not affect JNK phosphorylation following SE. Considering that EGCG suppresses JNK phosphorylation [69,70], our findings suggested that the neuroprotective mechanism of EGCG against oxidative stress-induced cell death included ROS–GPx1–ERK1/2-mediated DRP1 S616 phosphorylation, independent of JNK, unlike CDDO-Me.

NF-κB activation contributes to inflammatory reactions and oxidative stress after brain injuries and strokes [71,72]. Furthermore, oxidative stress further activates the NF-κB signaling pathway [73]. Following SE, NF-κB S536 phosphorylation enhanced CA1 and CA3 pyramidal cells vulnerable to SE [48,49]. Consistent with these reports, the present study revealed that SE elicited NF-κB S536 phosphorylation in CA1 neurons. In addition, EGCG diminished SE-induced NF-κB S536 phosphorylation, accompanied by GPx1 upregulation. Considering that the deletion or inhibition of GPx1 increases NF-κB S536 phosphorylation [56,57], it is likely that the antioxidant properties of EGCG may also attenuate SE-induced CA1 neuronal damage through NF-κB inhibition. Similar to EGCG, *N*-acetylcysteine (NAC, an antioxidant and GSH precursor) inhibits NF-κB S536 phosphorylation [74] and increases GPx1 expression in CA1 neurons following SE [34]. Although NF-κB mediated the activation of DRP1-dependent mitochondrial fission [54,55], the present study revealed SE-induced aberrant mitochondrial elongation concomitant with enhanced NF-κB S536 phosphorylation. Therefore, our findings suggested that EGCG may also mitigate SE-induced CA1 neuronal degeneration by abrogating the ROS–GPx1–NF-κB signaling pathway, which would not be involved in DRP1-mediated mitochondrial dynamics.

EGCG is known as an activator of non-integrin 67 kDa laminin receptor (67LR) that mediates ROS generation via the activation of NADPH oxidase [31]. However, the neuronal 67LR expression level is undetectable in the rat brain under physiological and post-SE conditions. Furthermore, 67LR neutralization cannot influence the p-ERK1/2 level in CA1 neurons, but diminishes it in astrocytes [75]. Therefore, it is likely that the antioxidant and neuroprotective effects of EGCG may not be mediated by 67LR.

The limitations of the present study were that we could not investigate (1) the molecular basis of hypoxic hippocampal neuronal damage induced by SE and (2) the permeability of EGCG across the brain–blood barrier (BBB). Although we could not find that animals ceased air intake during convulsions during the experimental procedures, the possibility that hypoxia (without apnea) could cause hippocampal neuronal damage could not be excluded. This is because ischemic–hypoxic lesions occur in the hippocampus during SE [76], and hypoxia disrupts mitochondrial dynamics in neurons [77]. Considering that short hypoxic preconditioning preceding SE promotes long-lasting protective effects on neuron survival and spatial memory [78], it is, however, an interesting topic to elucidate the effects of EGCG on hypoxia-related events during SE in the near future.

On the other hand, we applied an intracerebroventricular administration of EGCG in the present study because EGCG is poorly permeable to the BBB in rats [79]. Much of orally ingested EGCG is hydrolyzed to epigallocatechin (EGC) and gallic acid. Among them, EGC is catalyzed to 5-(3′,5′-dihydroxyphenyl)-γ-valerolactone (EGC-M5) and its conjugated forms, which are permeable to the BBB in vitro [80]. However, the permeability of EGCG into the brain is very low in vivo [79]. Of interest, Pervin et al. [81] inferred that the ingestion of EGCG (20 mg/kg) every day reached a concentration of ~0.6 µM in plasma and ~0.03 µM in the brain, which improved the cognitive function in mice. In rats, EGCG (100 mg/day for 4 weeks, intragastrically) could not penetrate into the brain tissue of control young rats, but was permeable to the BBB in aging rats with cognitive impairments due to the increased permeability of the BBB [79]. With respect to these reports, it is likely that EGCG may be permeable to the BBB under some pathophysiological conditions evoking increased BBB permeability or BBB disruptions such as SAH [28] and SE [23].

Recently, it was reported that the overexpression of mitofusin 2 (Mfn2, a protein regulating mitochondrial fusion) in neurons inhibited the lipopolysaccharide (LPS)-induced release of IL-1β but not TNF-α in the brain without affecting peripheral inflammation [82]. Regarding that EGCG protects hippocampal neurons against SE by inhibiting the Toll-like receptor 4–NF-κB–interleukin-1β (IL-1β) axis [29], the possibility that the EGCG-mediated restoration of mitochondrial dynamics in CA1 neurons would affect IL-1β synthesis following SE cannot be excluded. Therefore, it is presumable that impaired mitochondrial dynamics in neurons may be one of the underlying mechanisms regulating neuroinflammation, which could be mitigated by EGCG. Further studies are needed to elucidate this hypothesis.

## 5. Conclusions

**Figure 11:**
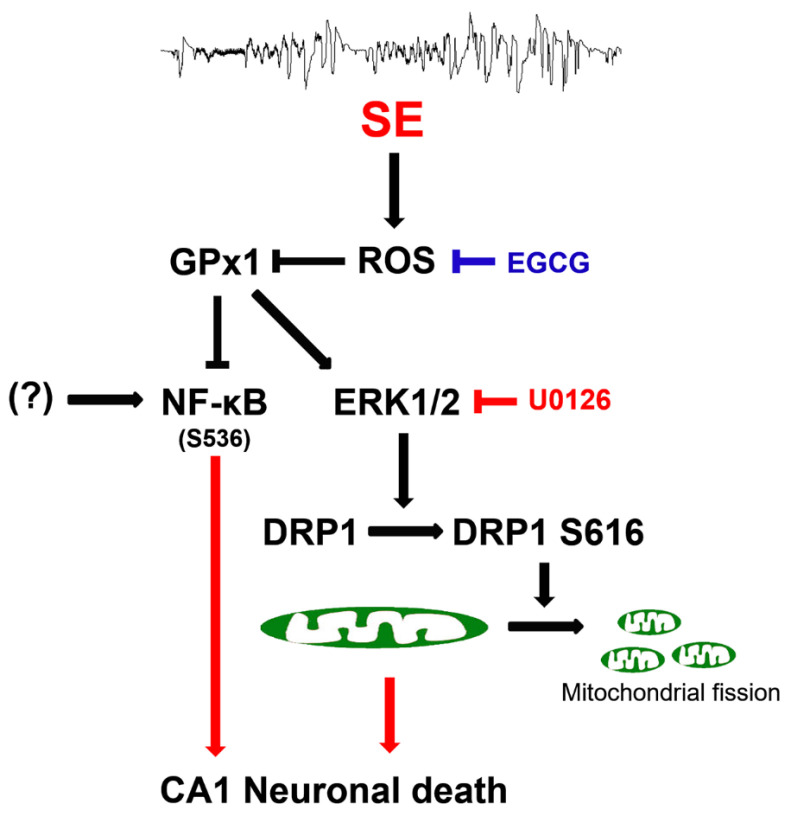
Scheme of the underlying mechanisms of the neuroprotective effects of EGCG against SE. EGCG abrogated SE-induced ROS generation, leading to GPx1 downregulation, which restored ERK1/2–DRP1-mediated mitochondrial fission and inhibited NF-κB S536 phosphorylation. These findings indicate that EGCG may protect CA1 neurons from SE insults through GPx1–ERK1/2–DRP1 and GPx1–NF-κB signaling pathways.

The present data showed that EGCG exerted GPx1 induction following SE, which attenuated CA1 neuronal death through the preservation of ERK1/2–DRP1-mediated mitochondrial fission, independent of JNK activity. EGCG also abolished the increment of NF-κB S536 phosphorylation in CA1 neurons following SE. These findings indicated that EGCG may protect CA1 neurons from SE insults through the ROS–GPx1–ERK1/2–DRP1 and ROS–GPx1–NF-κB signaling pathways. Therefore, our findings represent a future pharmacologic possibility that goes beyond the well-known antioxidative properties of EGCG (Figure 11).

## Figures and Tables

**Table 1:** Primary antibodies used in the present study.

Antigen	Hose	Manufacturer (Catalog Number)	Dilution
DRP1	Rabbit	Thermo (PA1-16987)	1:500 (IH) 1:1000 (WB)
ERK1/2	Rabbit	Biorbyt (Orb160960)	1:2000 (WB)
GPx1	Sheep	Biosensis (S-072-100)	1:2000 (IH) 1:10,000 (WB)
JNK	Rabbit	Proteintech (10023-1-AP)	1:1000 (WB)
Mitochondrial marker (Mitochondrial complex IV subunit 1, MTCO1)	Mouse	Abcam (#ab14705)	1:500 (IH)
NeuN	Guinea pig	Millipore (#ABN90P)	1:1000 (IH)
NF-κB S536	Rabbit	Abcam (#ab28856)	1:100 (IH)
p-DRP1 S616	Rabbit	Cell Signaling (#4494)	1:1000 (WB)
p-ERK1/2	Rabbit	Millipore (#05-797R)	1:100 (IH) 1:1000 (WB)
p-JNK	Rabbit	Millipore (#07-175)	1:1000 (WB)
β-actin	Mouse	Sigma (A5316)	1:5000 (WB)

WB: Western blot; IH: immunohistochemistry.

## Data Availability

The data are contained within the article and supplementary materials.

## Supplementary Materials

The following supporting information can be downloaded at https://www.mdpi.com/article/10.3390/antiox12040966/s1. Figure S1: Full-length gel images of Western blot data in Figure 3A. Figure S2: Full-length gel images of Western blot data in Figure 4A. Figure S3: Full-length gel images of Western blot data in Figure 6A. Figure S4: Full-length gel images of Western blot data in Figure 8A.
