# Supplementary material for: EGCG Attenuates CA1 Neuronal Death by Regulating GPx1, NF-κB S536 Phosphorylation and Mitochondrial Dynamics in the Rat Hippocampus following Status Epilepticus
Source: Antioxidants (Basel). 2023 Apr 20;12(4):966. doi: 10.3390/antiox12040966 (PMC10136286; doi:10.3390/antiox12040966)
Supplement: Supplementary file 1 [file antioxidants-12-00966-s001.zip › antioxidants-2309111-supplementary.pdf]

Supplementary Information

EGCG attenuates CA1 neuronal death by regulating GPx1, NF-κB S536 phosphorylation and mitochondrial dynamics in the rat hippocampus following status epilepticus

Ji-Eun Kim,[1] Tae-Hyun Kim,[1] Tae-Cheon Kang[1,*]

[1]Department of Anatomy and Neurobiology, Institute of Epilepsy Research, College of Medicine, Hallym University, Chuncheon 24252, South Korea

* Correspondence to: T. -C. Kang, Department of Anatomy and Neurobiology, College of Medicine, Hallym University, Chuncheon, Kangwon-Do 24252, South Korea; Tel: +82-33-248-2524; Fax: +82-33-248-2525; E-mail: tckang@hallym.ac.kr

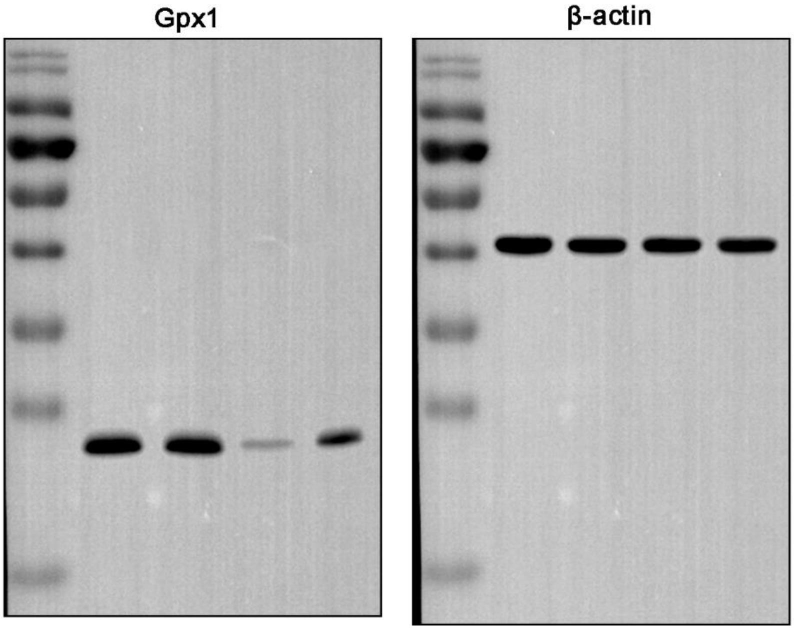

Supplementary Figure 1. Full-length gel images of Western blots in Figure 3A.

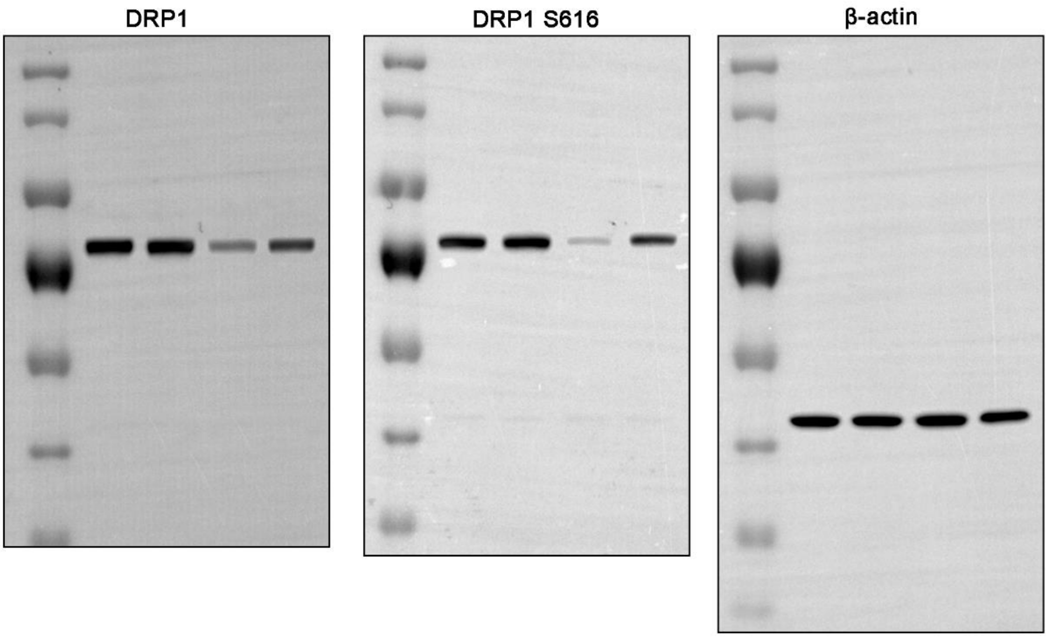

Supplementary Figure 2. Full-length gel images of Western blots in Figure 4A.

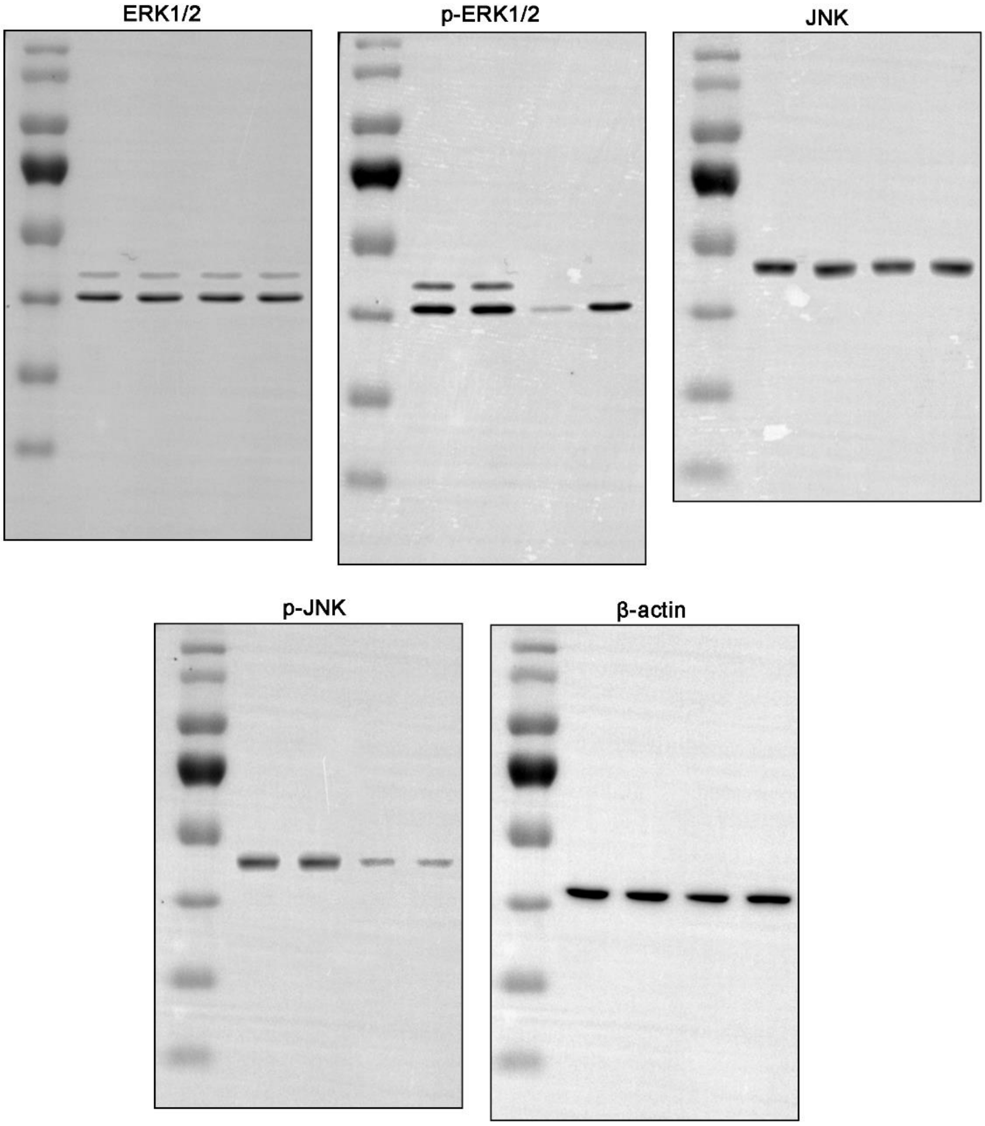

Supplementary Figure 3. Full-length gel images of Western blots in Figure 5A.

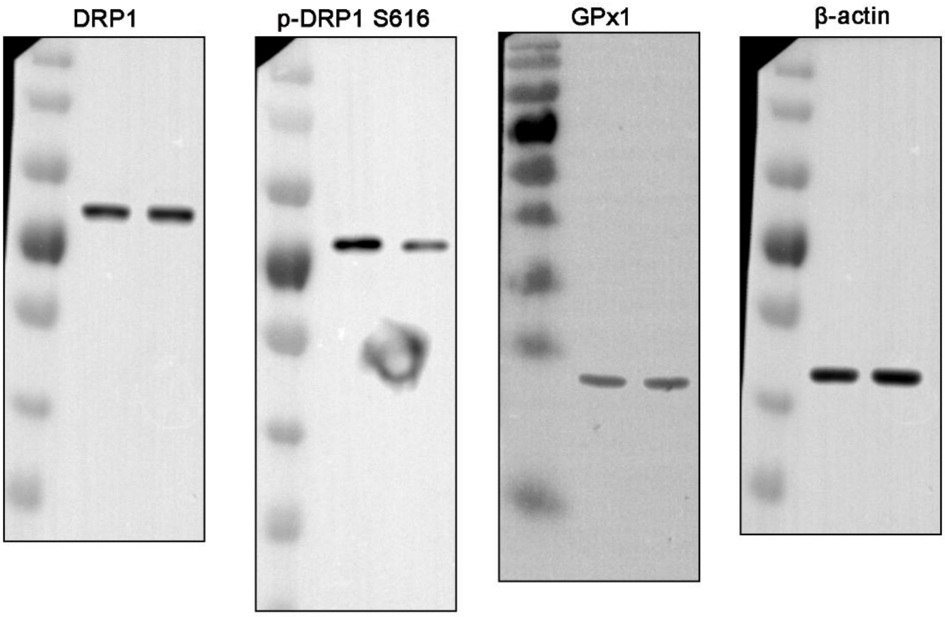

Supplementary Figure 4. Full-length gel images of Western blots in Figure 10A.
